# Supplementary material for: Metabolomics Analysis of DRG and Serum in the CCI Model of Mice
Source: Brain Sci. 2023 Aug 21;13(8):1224. doi: 10.3390/brainsci13081224 (PMC10452726; doi:10.3390/brainsci13081224)
Supplement: Supplementary file 1 [file brainsci-13-01224-s001.zip › brainsci-2533599-supplementary.pdf]

supplementary

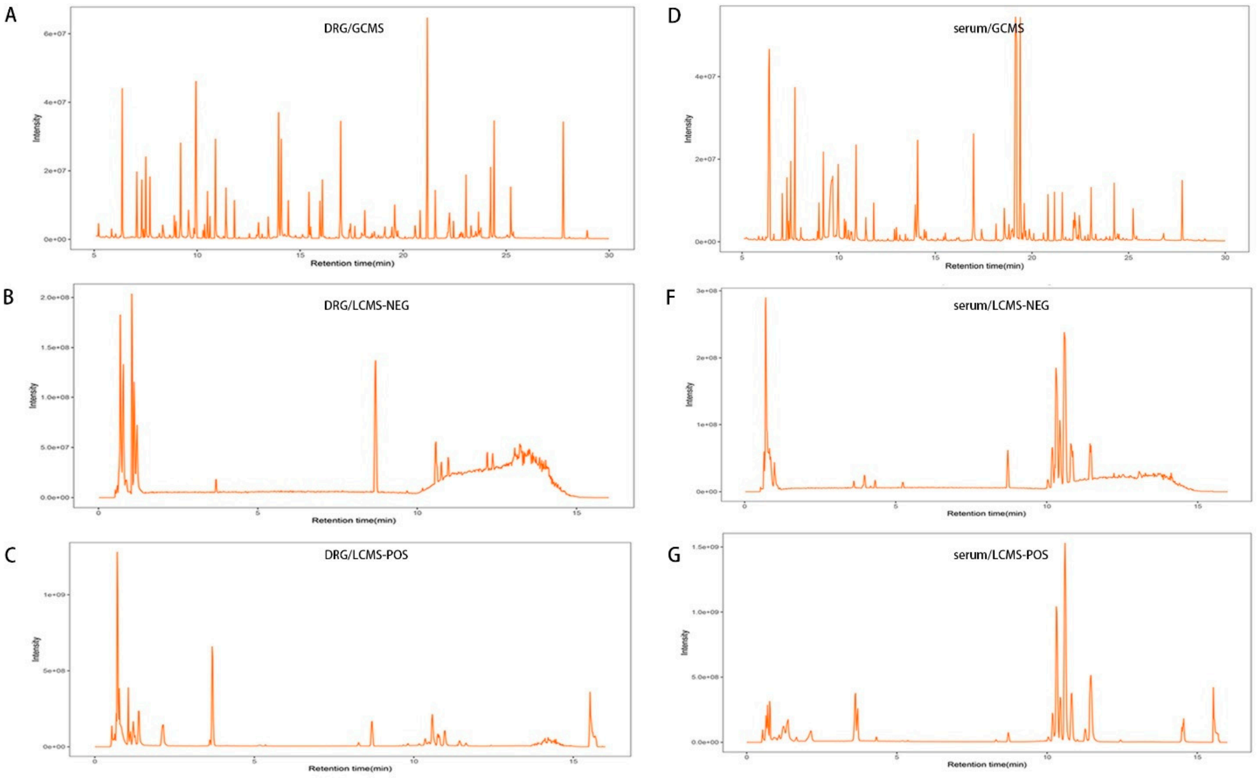

Figure S1. Total ion chromatogram of DRG extract acquired by GC-MS (A). The base peak chromatograms (BPIs) of DRG extract acquired by LC-MS in the positive (B) and negative (C) ion modes. Total ion chromatogram of serum extract acquired by GC-MS (D). The base peak chromatograms (BPIs) of serum extract acquired by LC-MS in the positive (F) and negative (G) ion modes.
